# Supplementary material for: Multi‐dataset identification of innovative feature genes and molecular mechanisms in keratoconus
Source: J Cell Mol Med. 2024 Sep 19;28(18):e70079. doi: 10.1111/jcmm.70079 (PMC11412914; doi:10.1111/jcmm.70079)
Supplement: Supplementary file 2 — Material S2. [file JCMM-28-e70079-s002.zip › 7.GSEA analysis/6.SNORA74B/KEGG_CHEMOKINE_SIGNALING_PATHWAY.html]

Details for gene set KEGG\_CHEMOKINE\_SIGNALING\_PATHWAY[GSEA]

|  || Dataset | SNORA74B.group.cls#h\_versus\_l |
| Phenotype | group.cls#h\_versus\_l |
| Upregulated in class | l |
| GeneSet | KEGG\_CHEMOKINE\_SIGNALING\_PATHWAY |
| Enrichment Score (ES) | -0.4661328 |
| Normalized Enrichment Score (NES) | -1.6337134 |
| Nominal p-value | 0.001996008 |
| FDR q-value | 0.30378905 |
| FWER p-Value | 0.545 |
Table: GSEA Results Summary

  

Fig 1: Enrichment plot: KEGG\_CHEMOKINE\_SIGNALING\_PATHWAY      
 Profile of the Running ES Score & Positions of GeneSet Members on the Rank Ordered List

  

| SYMBOL | TITLE | RANK IN GENE LIST | RANK METRIC SCORE | RUNNING ES | CORE ENRICHMENT || 1 | PIK3R5 | na | 170 | 0.800 | 0.0213 | No |
| 2 | PF4 | na | 941 | 0.497 | 0.0153 | No |
| 3 | ADCY2 | na | 2866 | 0.309 | -0.0303 | No |
| 4 | HCK | na | 3040 | 0.298 | -0.0256 | No |
| 5 | CCR10 | na | 4891 | 0.229 | -0.0716 | No |
| 6 | CXCR2 | na | 5077 | 0.223 | -0.0697 | No |
| 7 | CCR6 | na | 5158 | 0.220 | -0.0648 | No |
| 8 | JAK3 | na | 5304 | 0.215 | -0.0620 | No |
| 9 | BCAR1 | na | 5506 | 0.208 | -0.0610 | No |
| 10 | SOS1 | na | 5971 | 0.193 | -0.0681 | No |
| 11 | ADCY5 | na | 6025 | 0.192 | -0.0634 | No |
| 12 | ADCY7 | na | 6070 | 0.190 | -0.0584 | No |
| 13 | ITK | na | 6146 | 0.188 | -0.0545 | No |
| 14 | RASGRP2 | na | 7019 | 0.162 | -0.0744 | No |
| 15 | ADCY4 | na | 7191 | 0.157 | -0.0742 | No |
| 16 | GNAI3 | na | 7691 | 0.144 | -0.0840 | No |
| 17 | BRAF | na | 8089 | 0.134 | -0.0911 | No |
| 18 | PIK3R1 | na | 8116 | 0.133 | -0.0875 | No |
| 19 | CCR3 | na | 8240 | 0.131 | -0.0868 | No |
| 20 | CCL17 | na | 8406 | 0.127 | -0.0874 | No |
| 21 | PPBP | na | 8715 | 0.120 | -0.0924 | No |
| 22 | GNG8 | na | 8752 | 0.119 | -0.0895 | No |
| 23 | IKBKB | na | 8763 | 0.119 | -0.0859 | No |
| 24 | ROCK1 | na | 8851 | 0.117 | -0.0846 | No |
| 25 | ARRB1 | na | 9230 | 0.109 | -0.0920 | No |
| 26 | PLCB3 | na | 9334 | 0.106 | -0.0915 | No |
| 27 | AKT2 | na | 9380 | 0.105 | -0.0894 | No |
| 28 | PLCB2 | na | 9573 | 0.101 | -0.0916 | No |
| 29 | KRAS | na | 9766 | 0.097 | -0.0940 | No |
| 30 | CCL15 | na | 9912 | 0.094 | -0.0951 | No |
| 31 | VAV2 | na | 10064 | 0.091 | -0.0965 | No |
| 32 | CCR4 | na | 10538 | 0.083 | -0.1075 | No |
| 33 | ADCY9 | na | 10911 | 0.076 | -0.1158 | No |
| 34 | ADCY1 | na | 11165 | 0.072 | -0.1207 | No |
| 35 | CCL23 | na | 11184 | 0.072 | -0.1189 | No |
| 36 | PRKCZ | na | 12067 | 0.059 | -0.1425 | No |
| 37 | CXCR5 | na | 12403 | 0.055 | -0.1504 | No |
| 38 | ADCY6 | na | 12561 | 0.052 | -0.1532 | No |
| 39 | PF4V1 | na | 12586 | 0.052 | -0.1522 | No |
| 40 | PIK3R2 | na | 12946 | 0.047 | -0.1611 | No |
| 41 | PIK3CA | na | 13079 | 0.046 | -0.1634 | No |
| 42 | SOS2 | na | 13685 | 0.039 | -0.1796 | No |
| 43 | CCL11 | na | 13754 | 0.039 | -0.1803 | No |
| 44 | CSK | na | 14025 | 0.035 | -0.1870 | No |
| 45 | GNAI2 | na | 14445 | 0.031 | -0.1981 | No |
| 46 | STAT1 | na | 14481 | 0.030 | -0.1981 | No |
| 47 | XCL1 | na | 14545 | 0.030 | -0.1989 | No |
| 48 | CCL4L2 | na | 15123 | 0.023 | -0.2149 | No |
| 49 | PARD3 | na | 15192 | 0.023 | -0.2161 | No |
| 50 | STAT5B | na | 15213 | 0.022 | -0.2160 | No |
| 51 | PIK3CB | na | 15769 | 0.017 | -0.2315 | No |
| 52 | ARRB2 | na | 15989 | 0.015 | -0.2373 | No |
| 53 | VAV3 | na | 16097 | 0.014 | -0.2400 | No |
| 54 | TIAM1 | na | 16272 | 0.012 | -0.2446 | No |
| 55 | CHUK | na | 16402 | 0.010 | -0.2480 | No |
| 56 | WAS | na | 16570 | 0.009 | -0.2526 | No |
| 57 | XCR1 | na | 16622 | 0.008 | -0.2538 | No |
| 58 | MAPK3 | na | 16753 | 0.007 | -0.2573 | No |
| 59 | PLCB4 | na | 16838 | 0.006 | -0.2596 | No |
| 60 | PRKCD | na | 17017 | 0.005 | -0.2646 | No |
| 61 | CCL18 | na | 17170 | 0.003 | -0.2689 | No |
| 62 | PTK2B | na | 17452 | 0.000 | -0.2770 | No |
| 63 | CCL1 | na | 17639 | -0.002 | -0.2823 | No |
| 64 | ROCK2 | na | 17655 | -0.002 | -0.2827 | No |
| 65 | GNB2 | na | 17690 | -0.003 | -0.2836 | No |
| 66 | WASL | na | 17732 | -0.003 | -0.2847 | No |
| 67 | RAP1A | na | 17815 | -0.004 | -0.2869 | No |
| 68 | GSK3B | na | 17871 | -0.004 | -0.2884 | No |
| 69 | RHOA | na | 18290 | -0.009 | -0.3001 | No |
| 70 | CCL26 | na | 18307 | -0.010 | -0.3003 | No |
| 71 | AKT1 | na | 18366 | -0.010 | -0.3016 | No |
| 72 | STAT2 | na | 18472 | -0.012 | -0.3043 | No |
| 73 | GNG4 | na | 18523 | -0.012 | -0.3054 | No |
| 74 | RAP1B | na | 18593 | -0.013 | -0.3069 | No |
| 75 | GSK3A | na | 18632 | -0.013 | -0.3076 | No |
| 76 | ADCY8 | na | 18678 | -0.014 | -0.3085 | No |
| 77 | CDC42 | na | 18770 | -0.015 | -0.3106 | No |
| 78 | PIK3CG | na | 18884 | -0.016 | -0.3134 | No |
| 79 | RAC2 | na | 18892 | -0.016 | -0.3131 | No |
| 80 | CXCL5 | na | 19002 | -0.017 | -0.3157 | No |
| 81 | GNG7 | na | 19060 | -0.018 | -0.3167 | No |
| 82 | CCL24 | na | 19163 | -0.019 | -0.3191 | No |
| 83 | GNG10 | na | 19308 | -0.021 | -0.3225 | No |
| 84 | CCL16 | na | 19397 | -0.022 | -0.3244 | No |
| 85 | HRAS | na | 19699 | -0.025 | -0.3323 | No |
| 86 | GRK6 | na | 19851 | -0.027 | -0.3358 | No |
| 87 | RAC1 | na | 19881 | -0.027 | -0.3357 | No |
| 88 | SHC3 | na | 20230 | -0.031 | -0.3448 | No |
| 89 | PIK3CD | na | 20354 | -0.032 | -0.3473 | No |
| 90 | MAPK1 | na | 20606 | -0.035 | -0.3534 | No |
| 91 | CCL8 | na | 20722 | -0.037 | -0.3555 | No |
| 92 | IKBKG | na | 20820 | -0.038 | -0.3571 | No |
| 93 | GNG5 | na | 21032 | -0.040 | -0.3619 | No |
| 94 | RAF1 | na | 21074 | -0.041 | -0.3617 | No |
| 95 | PRKX | na | 21076 | -0.041 | -0.3604 | No |
| 96 | CCL27 | na | 21088 | -0.041 | -0.3594 | No |
| 97 | GRK5 | na | 21299 | -0.044 | -0.3640 | No |
| 98 | CXCL14 | na | 21482 | -0.046 | -0.3678 | No |
| 99 | CCL19 | na | 21660 | -0.048 | -0.3714 | No |
| 100 | GNG13 | na | 21847 | -0.050 | -0.3751 | No |
| 101 | NFKBIB | na | 22042 | -0.052 | -0.3790 | No |
| 102 | GNB1 | na | 22177 | -0.054 | -0.3812 | No |
| 103 | CXCL16 | na | 22313 | -0.056 | -0.3832 | No |
| 104 | CCR1 | na | 22388 | -0.057 | -0.3835 | No |
| 105 | PRKACA | na | 22659 | -0.060 | -0.3894 | No |
| 106 | PTK2 | na | 22693 | -0.061 | -0.3883 | No |
| 107 | SHC1 | na | 22752 | -0.062 | -0.3880 | No |
| 108 | FOXO3 | na | 22782 | -0.062 | -0.3868 | No |
| 109 | CCR8 | na | 22954 | -0.065 | -0.3897 | No |
| 110 | CCR5 | na | 23558 | -0.074 | -0.4047 | No |
| 111 | GNG3 | na | 23659 | -0.075 | -0.4051 | No |
| 112 | CXCL12 | na | 23758 | -0.077 | -0.4054 | No |
| 113 | GNG2 | na | 23916 | -0.079 | -0.4074 | No |
| 114 | GNG12 | na | 23995 | -0.081 | -0.4070 | No |
| 115 | TIAM2 | na | 24277 | -0.085 | -0.4123 | No |
| 116 | JAK2 | na | 24375 | -0.087 | -0.4123 | No |
| 117 | GRB2 | na | 24997 | -0.098 | -0.4271 | No |
| 118 | PXN | na | 26060 | -0.118 | -0.4539 | No |
| 119 | STAT3 | na | 26362 | -0.124 | -0.4586 | No |
| 120 | CXCL13 | na | 26404 | -0.125 | -0.4557 | No |
| 121 | NFKB1 | na | 26534 | -0.128 | -0.4552 | No |
| 122 | CCR2 | na | 26723 | -0.133 | -0.4563 | No |
| 123 | CRK | na | 26740 | -0.134 | -0.4524 | No |
| 124 | ADCY3 | na | 26777 | -0.134 | -0.4490 | No |
| 125 | GNB5 | na | 26824 | -0.136 | -0.4459 | No |
| 126 | CCL14 | na | 27083 | -0.141 | -0.4488 | No |
| 127 | NCF1 | na | 27684 | -0.156 | -0.4610 | Yes |
| 128 | CCL13 | na | 27846 | -0.161 | -0.4604 | Yes |
| 129 | CXCL11 | na | 27958 | -0.164 | -0.4583 | Yes |
| 130 | NRAS | na | 27969 | -0.164 | -0.4532 | Yes |
| 131 | CCL3L3 | na | 28062 | -0.166 | -0.4504 | Yes |
| 132 | CCL28 | na | 28142 | -0.169 | -0.4472 | Yes |
| 133 | GNAI1 | na | 28399 | -0.176 | -0.4488 | Yes |
| 134 | PAK1 | na | 28478 | -0.179 | -0.4452 | Yes |
| 135 | CCL2 | na | 28523 | -0.180 | -0.4406 | Yes |
| 136 | NFKBIA | na | 28570 | -0.182 | -0.4360 | Yes |
| 137 | CXCL2 | na | 28621 | -0.183 | -0.4315 | Yes |
| 138 | CCR9 | na | 29240 | -0.202 | -0.4428 | Yes |
| 139 | CRKL | na | 29271 | -0.203 | -0.4370 | Yes |
| 140 | LYN | na | 29275 | -0.203 | -0.4304 | Yes |
| 141 | PIK3R3 | na | 29453 | -0.208 | -0.4287 | Yes |
| 142 | CXCL6 | na | 29519 | -0.210 | -0.4238 | Yes |
| 143 | FGR | na | 29584 | -0.212 | -0.4187 | Yes |
| 144 | RELA | na | 29651 | -0.214 | -0.4136 | Yes |
| 145 | GNGT2 | na | 29687 | -0.215 | -0.4075 | Yes |
| 146 | CCL21 | na | 30451 | -0.243 | -0.4217 | Yes |
| 147 | CCL7 | na | 30636 | -0.250 | -0.4188 | Yes |
| 148 | GRK4 | na | 30718 | -0.254 | -0.4128 | Yes |
| 149 | ELMO1 | na | 30779 | -0.257 | -0.4062 | Yes |
| 150 | GNB3 | na | 30930 | -0.264 | -0.4019 | Yes |
| 151 | CXCR6 | na | 30944 | -0.265 | -0.3936 | Yes |
| 152 | MAP2K1 | na | 31129 | -0.275 | -0.3899 | Yes |
| 153 | CXCL8 | na | 31337 | -0.285 | -0.3866 | Yes |
| 154 | CXCR3 | na | 31747 | -0.309 | -0.3883 | Yes |
| 155 | CCL22 | na | 31926 | -0.319 | -0.3830 | Yes |
| 156 | CCL5 | na | 32237 | -0.337 | -0.3810 | Yes |
| 157 | CCL3 | na | 32264 | -0.338 | -0.3707 | Yes |
| 158 | PRKACB | na | 32349 | -0.344 | -0.3618 | Yes |
| 159 | CCL20 | na | 32374 | -0.346 | -0.3512 | Yes |
| 160 | SHC4 | na | 32485 | -0.353 | -0.3429 | Yes |
| 161 | PREX1 | na | 32696 | -0.368 | -0.3369 | Yes |
| 162 | PRKACG | na | 32698 | -0.368 | -0.3249 | Yes |
| 163 | PLCB1 | na | 32854 | -0.381 | -0.3169 | Yes |
| 164 | GRK7 | na | 32883 | -0.384 | -0.3052 | Yes |
| 165 | VAV1 | na | 32928 | -0.388 | -0.2938 | Yes |
| 166 | GRK1 | na | 33143 | -0.408 | -0.2866 | Yes |
| 167 | CX3CR1 | na | 33225 | -0.415 | -0.2754 | Yes |
| 168 | GNGT1 | na | 33266 | -0.421 | -0.2627 | Yes |
| 169 | CXCL3 | na | 33497 | -0.449 | -0.2547 | Yes |
| 170 | CXCR4 | na | 33720 | -0.481 | -0.2454 | Yes |
| 171 | CCR7 | na | 33758 | -0.486 | -0.2305 | Yes |
| 172 | DOCK2 | na | 33838 | -0.501 | -0.2164 | Yes |
| 173 | CX3CL1 | na | 33846 | -0.503 | -0.2001 | Yes |
| 174 | PRKCB | na | 33882 | -0.508 | -0.1845 | Yes |
| 175 | CCL25 | na | 34109 | -0.559 | -0.1728 | Yes |
| 176 | GNG11 | na | 34191 | -0.579 | -0.1562 | Yes |
| 177 | GNB4 | na | 34205 | -0.582 | -0.1375 | Yes |
| 178 | SHC2 | na | 34407 | -0.652 | -0.1220 | Yes |
| 179 | CXCR1 | na | 34414 | -0.656 | -0.1007 | Yes |
| 180 | CXCL1 | na | 34497 | -0.699 | -0.0802 | Yes |
| 181 | CXCL9 | na | 34585 | -0.776 | -0.0573 | Yes |
| 182 | AKT3 | na | 34657 | -0.880 | -0.0306 | Yes |
| 183 | CXCL10 | na | 34695 | -0.997 | 0.0010 | Yes |
Table: GSEA details [plain text format]

  

Fig 2: KEGG\_CHEMOKINE\_SIGNALING\_PATHWAY      
 Blue-Pink O' Gram in the Space of the Analyzed GeneSet

  

Fig 3: KEGG\_CHEMOKINE\_SIGNALING\_PATHWAY: Random ES distribution      
 Gene set null distribution of ES for **KEGG\_CHEMOKINE\_SIGNALING\_PATHWAY**
